# Exploratory associations between radiographic findings and metadata-derived proxies of 90-day follow-up in 112,120 ChestX-ray14 radiographs

**DOI:** 10.1038/s41598-025-31885-3

**Published:** 2025-12-09

**Authors:** Josef Yayan, Kurt Rasche, Marcus Krüger, Christian Biancosino

**Affiliations:** 1 Department of Internal Medicine, Division of Pulmonary, Allergy, and Sleep Medicine, HELIOS Clinic Wuppertal, Witten/Herdecke University, Witten, Germany; 2 Department of Thoracic Surgery, Martha-Maria Hospital Halle-Dölau, Halle, Germany; 3 Department of Thoracic Surgery, HELIOS Clinic Wuppertal, Witten/Herdecke University, Witten, Germany; 4 Department of Internal Medicine, Division of Pulmonary, Allergy, and Sleep Medicine, HELIOS Clinic Wuppertal Witten/Herdecke University, Heusnerstr. 40, 42283 Wuppertal, Germany

**Keywords:** Chest radiography, ChestX-ray14 (NIH dataset), Metadata-derived proxy outcome, Exploratory analysis, Sex differences, Radiographic findings, Logistic regression, Diseases, Health care, Medical research, Risk factors

## Abstract

Chest radiography is widely used as an initial imaging modality. However, how specific findings relate to subsequent care or follow-up actions remains unclear. Prior studies have rarely examined follow-up actions, and potential sex-specific differences have been understudied. We analyzed 112,120 frontal chest radiographs from the NIH ChestX-ray14 dataset (63,340 male, 48,780 female). Images were labeled with 14 findings using a natural language processing (NLP) pipeline applied to reports. We modeled a metadata-derived proxy of 90-day follow-up using logistic regression, including sex-stratified analyses and interaction testing, with models adjusted for sex. Robustness was assessed through sensitivity analyses (30/60/180-day windows), patient-level clustering, and false discovery rate (FDR) adjustment. The strongest associations with proxy follow-up were observed for pulmonary edema (OR 10.6, 95% CI 8.5–13.2), pneumothorax (OR 7.6, 95% CI 6.7–8.6), and pleural effusion (OR 4.0, 95% CI 3.8–4.3). Interactions between sex and specific findings were modest but reached statistical significance for atelectasis (*P* = 0.003), pneumothorax (*P* = 0.0083), and emphysema (*P* = 0.0238). Radiographic findings were associated with metadata-derived proxy follow-up, but residual confounding from unmeasured factors (e.g., age, comorbidities, clinical context) likely remains. Sex-specific effects were statistically significant but small, and not clinically meaningful. Results should therefore be interpreted strictly as hypothesis-generating signals, not as causal evidence or clinically directive guidance.

**Supplementary Information:**

The online version contains supplementary material available at 10.1038/s41598-025-31885-3.

## Introduction

Chest radiography is one of the most widely used imaging modalities and primarily serves as an initial rather than a comprehensive diagnostic test for thoracic pathology^1^. Despite limited sensitivity for certain conditions, it remains essential due to its broad widespread availability, rapid acquisition, and role in triage, as emphasized in international guidelines^2^. Radiological findings frequently guide subsequent diagnostic work-up or treatment initiation^3^.

With the rise of large-scale datasets and machine learning applications, automated labeling of radiological images has gained importance^4^. One of the most influential resources is the ChestX-ray14 database, which provides labeled frontal chest radiographs derived from natural language processing (NLP) of reports^5^. While enabling large-scale research, this approach introduces label noise and misclassification, as the accuracy of NLP-derived labels varies substantially across findings (e.g., high for pneumothorax, lower for infiltration)^6^. Both the labels and the dataset’s follow-up field should therefore be regarded as proxies derived from metadata rather than validated clinical outcomes^7^.

Despite extensive use of ChestX-ray14 in diagnostic classification research, little is known about how specific radiographic findings relate to subsequent follow-up actions. Previous studies have focused mainly on image-based diagnostic accuracy, whereas the link between findings and patient-level care pathways has received limited attention^4–6^. The follow-up information provided in the dataset is a metadata-derived proxy that may capture both clinical and non-clinical events (e.g., routine or administrative imaging). Moreover, crucial covariates such as age, comorbidities, and inpatient versus outpatient care settings are absent, which restricts interpretability and increases the risk of residual confounding.

Sex-specific differences represent another understudied aspect. Follow-up likelihood may vary by biological variation, disease prevalence, or presentation, but may also reflect provider response and potential bias in clinical decision-making^8–11^.

In light of these gaps, the present study was explicitly designed as exploratory and descriptive. We aimed to evaluate the associations between specific radiographic findings and metadata-derived proxies of follow-up in ChestX-ray14, and to assess whether these associations differ between males and females through sex-stratified analyses and interaction testing. Given the absence of key covariates and reliance on metadata proxies, our results should be interpreted solely as hypothesis-generating signals; causal inference or clinical recommendations are beyond the scope of this analysis.

## Materials and methods

### Study design and objectives

This study employed a retrospective, cross-sectional design based on a large-scale dataset of chest radiographs from adult patients. The primary objective was to evaluate the association between specific radiological findings on frontal chest X-rays and the subsequent occurrence of metadata-derived proxy measures of clinical follow-up, acknowledging that this endpoint is not a validated patient-level action. A secondary objective was to assess sex-specific differences in these associations through stratified analyses and interaction testing. The study was explicitly designed as exploratory, with results intended to generate hypotheses rather than provide causal or clinically directive conclusions.

### Data source

All radiographic data were obtained from the publicly accessible ChestX-ray14 dataset, released by the National Institutes of Health (NIH). This dataset contains over 112,120 frontal chest radiographs collected from more than 30,000 unique patients between 1992 and 2015 at the NIH Clinical Center. As all data were derived from a single tertiary care institution, the generalizability to other healthcare settings is limited, and temporal changes in practice since 2015 (including the COVID-19 era) are not represented. For the purposes of this study, a curated subset comprising 112,120 radiographs was used, including 63,340 from male patients and 48,780 from female patients. Only radiographs with clearly defined sex (male or female) and valid radiological labels were included. Each image in the dataset was annotated with one or more radiological findings using a natural language processing (NLP) pipeline applied to corresponding radiology reports. The accuracy of these labels is known to vary substantially across findings (e.g., higher for pneumothorax, lower for infiltration)^4^. Accordingly, labels were treated as metadata-derived proxies rather than ground truth. To avoid confusion with earlier dataset versions, we consistently refer to ChestX-ray14 throughout this study. The dataset does not contain clinical covariates such as disease severity, comorbidities, or care settings, which restricts interpretability of follow-up associations. Occasional metadata artifacts, such as records indicating more than four radiographs per day or fractional encounter counts per patient, were retained as provided in the ChestX-ray14 dataset to preserve reproducibility, as detailed in Supplementary Figure B.

### Radiological findings

The study included the following 14 radiological findings: atelectasis, cardiomegaly, consolidation, edema, effusion, emphysema, fibrosis, hernia, infiltration, mass, nodule, pleural thickening, pneumonia, and pneumothorax. Radiographs without any of these abnormalities were classified as “No Finding.” To avoid misinterpretation, “No Finding” was used descriptively and as the reference category in regression models; it was not entered as an independent predictor. Each finding was treated as a binary variable indicating presence or absence. It was possible for a single image to be assigned multiple findings, reflecting the clinical reality of overlapping pathologies. Potential co-occurrence of findings was accounted for by including all 14 variables simultaneously in multivariable models.  However, no additional stratification or modeling of specific combinations of acute and chronic findings (e.g., pneumothorax with fibrosis) was performed, which may have influenced follow-up associations.

### Outcome definition

The primary outcome of interest was proxy follow-up, defined as any radiology encounter documented within 90 days after the index chest radiograph. This was operationalized using the dataset’s “follow-up” metadata field, which records whether additional radiographs or related examinations were obtained for the same patient within the specified time window. This field may reflect both clinical and non-clinical events (e.g., administrative or pre-scheduled imaging) and must therefore be interpreted as a proxy for downstream activity rather than a validated measure of radiograph-triggered follow-up. The variable was analyzed as a binary outcome (yes/no). Because the dataset does not distinguish between inpatient and outpatient settings, stratification by care setting was not possible. Temporality between radiographic findings and subsequent events could not be confirmed, and reverse causation cannot be excluded. The “follow-up” field is part of the official ChestX-ray14 release (Wang et al., 2017) and is described in the NIH dataset documentation (https://nihcc.app.box.com/v/ChestXray-NIHCC, accessed 7 October 2025).

### Statistical analysis

Descriptive statistics were used to report the prevalence of each radiological finding by sex. Associations between findings and proxy follow-up were modeled with logistic regression. In the primary model, all 14 findings were included as independent variables and adjusted for sex. Cluster-robust standard errors at the patient level were applied to account for repeated measures. Sensitivity analyses included: (i) restricting to the first radiograph per patient, (ii) varying the follow-up window to 30, 60, and 180 days, and (iii) applying a Benjamini–Hochberg false discovery rate (FDR) correction. To assess potential multicollinearity, variance inflation factors (VIFs) were calculated (all < 5). In addition, average marginal effects were estimated to enhance interpretability of associations. Odds ratios (ORs) with 95% confidence intervals (CIs) and P-values were reported. Sex-specific effects were evaluated using stratified models and interaction terms. All analyses were performed in R (version 4.3.2, R Foundation for Statistical Computing, Vienna, Austria). P-values are nominal and interpreted cautiously in this exploratory context; FDR adjustment was applied in supplementary analyses as a robustness check.

## Results

**Fig. 1:**
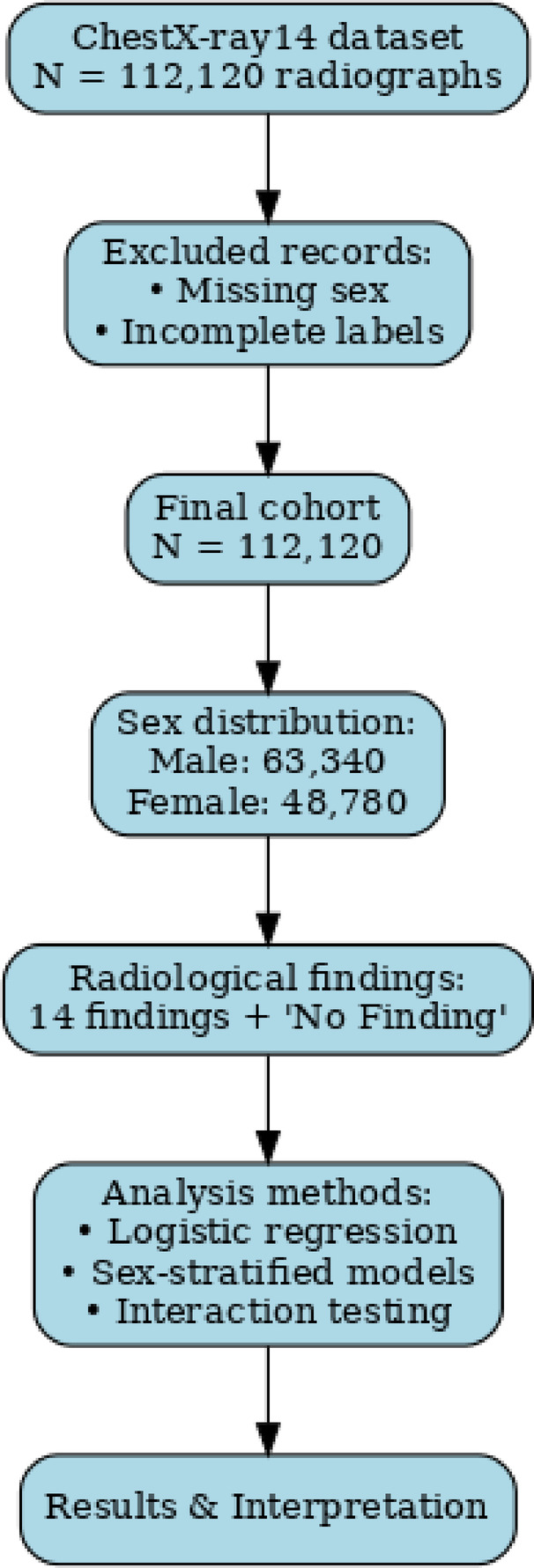
Study flowchart of patient and image selection from the ChestX-ray14 dataset. A total of 112,120 frontal chest radiographs were available. After excluding records with missing sex or incomplete labels, the final cohort included 63,340 male and 48,780 female radiographs. Each image was annotated with 14 radiological findings plus a “No Finding” category using an NLP pipeline. Follow-up was defined from metadata as a proxy outcome. No radiographs were excluded due to missing follow-up data.

This study analyzed a total of 112,120 chest radiographs from adult patients, including 63,340 from male and 48,780 from female patients (Table 1; Fig. 1). Follow-up metadata were complete, and no radiographs were excluded due to missing outcome information. Each radiograph was annotated with one or more of 14 predefined thoracic findings using an NLP-based labeling pipeline. The evaluated findings included pulmonary edema, pleural effusion, pneumothorax, consolidation, atelectasis, emphysema, pneumonia, cardiomegaly, infiltration, nodule, mass, pleural thickening, fibrosis, and diaphragmatic hernia. Radiographs without any detected abnormality were classified as “No Finding” and used descriptively as the reference category in regression models.

**Table 1:** Baseline characteristics of the study cohort. A total of 112,120 frontal chest radiographs were included, with 63,340 (56.5%) from male and 48,780 (43.5%) from female patients. On average, 0.72 ± 0.96 radiological findings were assigned per image, reflecting frequent co-occurrence of abnormalities. The most common findings were infiltration, pleural effusion, and atelectasis. Follow-up was defined as a metadata-derived proxy and operationalized as any recorded radiology encounter within 90 days of the index chest radiograph. No radiographs were excluded due to missing follow-up information.

Characteristic	Value
Total radiographs	112,120
Male patients	63,340 (56.5%)
Female patients	48,780 (43.5%)
Mean number of findings per image	0.72 ± 0.96
Most frequent findings	Infiltration, Effusion, Atelectasis
Follow-up ascertainment window	90 days (metadata-derived proxy)

Infiltration was the most frequent abnormality (*n* = 19,894; 17.7%), followed by pleural effusion (*n* = 13,317; 11.9%) and atelectasis (*n* = 11,559; 10.3%). By contrast, fibrosis (*n* = 1,686; 1.5%) and hernia (*n* = 227; 0.2%) were rare findings in the cohort.

As shown in Table 2, pulmonary edema (OR 10.6, 95% CI 8.5–13.2), pneumothorax (OR 7.6, 95% CI 6.7–8.6), and pleural effusion (OR 4.0, 95% CI 3.8–4.3) were most strongly associated with proxy follow-up (all *P* < 0.001). Consolidation (OR 3.9), emphysema (OR 3.3), pneumonia (OR 2.7), and atelectasis (OR 2.3) also showed significant associations (all *P* < 0.001). More modest associations were observed for infiltration (OR 1.9), mass (OR 1.3), and pleural thickening (OR 1.3). Cardiomegaly was not significantly associated with follow-up (OR 1.0, *P* = 0.93), while nodule showed only a slight increase (OR 1.1, *P* = 0.036). By contrast, fibrosis (OR 0.7, *P* < 0.001) and diaphragmatic hernia (OR 0.7, *P* = 0.0037) were associated with decreased odds of follow-up.

**Table 2:** Multivariable logistic regression of radiological findings and proxy follow-up within 90 days. Results of a logistic regression model including all 14 radiological findings simultaneously, adjusted for sex. Odds ratios (OR) with 95% confidence intervals (CI) and two-sided P-values are reported. Variance inflation factors (VIFs) ranged from 1.00 to 1.08, indicating limited collinearity. Statistically significant P-values (*P* < 0.05) are shown in bold. “No finding” served as the reference category and was not included as a predictor.

Finding	Male *N* = 63,340 (n)	Female *N* = 48,780 (n)	Odds Ratio	95% CI (Lower)	95% CI (upper)	*P*-value
Edema	1204	1099	10.6	8.5	13.2	**< 0.001**
Pneumothorax	2717	2585	7.6	6.7	8.6	**< 0.001**
Effusion	7435	5882	4.0	3.8	4.3	**< 0.001**
Consolidation	2666	2001	3.9	3.5	4.3	**< 0.001**
Emphysema	1610	906	3.3	2.9	3.7	**< 0.001**
Pneumonia	838	593	2.7	2.3	3.2	**< 0.001**
Atelectasis	6906	4653	2.3	2.2	2.5	**< 0.001**
Infiltration	11,427	8467	1.9	1.8	2.0	**< 0.001**
Mass	3529	2253	1.3	1.3	1.4	**< 0.001**
Pleural thickening	2042	1343	1.3	1.2	1.4	**< 0.001**
Nodule	3685	2646	1.1	1.0	1.1	**0.0357**
Cardiomegaly	1307	1469	1.0	0.9	1.1	0.9304
Fibrosis	915	771	0.7	0.7	0.8	**< 0.001**
Hernia	96	131	0.7	0.5	0.9	**0.0037**
No finding	33,922	26,439	–	–	–	–

Follow-up was defined using metadata as a proxy and should be interpreted cautiously. Radiological labels were NLP-derived and subject to variable accuracy (higher for pneumothorax, lower for infiltration [Wang et al.^4^]). “No Finding” served as the reference category and was not estimated. Results are exploratory and not definitive evidence of clinical practice.

Results remained consistent when restricting the analysis to the first radiograph per patient (Supplementary Table X).

Sex-stratified analyses (Table 3) were consistent with overall trends but also suggested differences in effect size. Pulmonary edema was more strongly associated with follow-up in females (OR 12.8, 95% CI 9.15–17.96) than in males (OR 9.0, 95% CI 6.69–12.0). Pneumothorax was more predictive of follow-up in males (OR 9.1) compared to females (OR 6.5). Similarly, the associations for emphysema (OR 4.0 vs. 2.9) and atelectasis (OR 2.6 vs. 2.2) were stronger in females. Other findings such as consolidation, pneumonia, mass, and infiltration showed broadly similar effect sizes across both sexes.

**Table 3:** Sex-stratified odds ratios for proxy clinical follow-up associated with radiological findings. Logistic regression models were fitted separately for male and female patients to evaluate the association between individual radiological findings and the likelihood of proxy follow-up. Each sex-stratified model included all 14 radiological findings concurrently, thereby accounting for co-occurrence; no sex covariate was included within strata. Odds ratios (ORs) greater than 1 indicate higher odds of follow-up, while ORs less than 1 indicate lower odds. Values are presented with 95% confidence intervals (CIs) and two-sided nominal P-values, reported without correction for multiple testing. Statistically significant P-values (*P* < 0.05) are highlighted in bold. Results are exploratory and should not be considered definitive evidence of clinical practice.

Finding	OR (male)	95% CI (male)	*P* (male)	OR (female)	95% CI (female)	*P* (female)
Pneumothorax	9.1	[7.53–11.1]	**< 0.001**	6.5	[5.55–7.64]	**< 0.001**
Edema	9.0	[6.69–12.0]	**< 0.001**	12.8	[9.15–17.96]	**< 0.001**
Effusion	4.0	[3.72–4.38]	**< 0.001**	4.0	[3.65–4.34]	**< 0.001**
Consolidation	3.8	[3.29–4.3]	**< 0.001**	4.1	[3.54–4.79]	**< 0.001**
Emphysema	2.9	[2.48–3.4]	**< 0.001**	4.0	[3.18–4.97]	**< 0.001**
Pneumonia	2.7	[2.15–3.27]	**< 0.001**	2.7	[2.15–3.47]	**< 0.001**
Atelectasis	2.2	[2.04–2.34]	**< 0.001**	2.6	[2.37–2.8]	**< 0.001**
Infiltration	1.8	[1.75–1.94]	**< 0.001**	1.9	[1.83–2.06]	**< 0.001**
Mass	1.3	[1.22–1.44]	**< 0.001**	1.4	[1.23–1.5]	**< 0.001**
Pleural thickening	1.3	[1.18–1.46]	**< 0.001**	1.3	[1.14–1.47]	**0.0001**
Nodule	1.1	[1.02–1.19]	**0.0143**	1.0	[0.93–1.11]	0.6951
Cardiomegaly	1.1	[0.93–1.2]	0.4034	1.0	[0.86–1.08]	0.5164
Fibrosis	0.7	[0.63–0.83]	**< 0.001**	0.8	[0.65–0.87]	**0.0002**
Hernia	0.6	[0.41–0.94]	**0.0243**	0.7	[0.5–1.01]	0.0568

Interaction analysis (Table 4) indicated statistically significant sex-specific effects for atelectasis (interaction OR 0.9, *P* = 0.003), pneumothorax (interaction OR 1.4, *P* = 0.0083), and emphysema (interaction OR 0.7, *P* = 0.0238). No significant interaction was found for infiltration, pleural effusion, pneumonia, or consolidation.

**Table 4:** Sex–finding interaction effects on the likelihood of proxy clinical follow-up. Logistic regression models included an interaction term between each radiological finding and sex (male vs. female). The “OR (Main Effect)” represents the main association pooled across sexes (reference: female), while the “OR (Interaction)” quantifies whether the association differs between males and females. Interaction ORs > 1 indicate a stronger effect in males; values < 1 indicate a stronger effect in females. Nominal P-values are reported without correction for multiple testing and should be interpreted as exploratory. Statistically significant P-values (*P* < 0.05) are highlighted in bold.

Finding	OR (main effect)	95% CI (main)	*P* (main)	OR (interaction)	95% CI (interaction)	*P* (interaction)
Atelectasis	2.6	[2.37–2.8]	**< 0.001**	0.9	[0.76–0.95]	**0.003**
Pneumothorax	6.5	[5.55–7.64]	**< 0.001**	1.4	[1.09–1.81]	**0.0083**
Emphysema	4.0	[3.18–4.97]	**< 0.001**	0.7	[0.56–0.96]	**0.0238**
Edema	12.8	[9.15–17.96]	**< 0.001**	0.7	[0.45–1.09]	0.1161
Nodule	1.0	[0.93–1.11]	0.6951	1.1	[0.96–1.22]	0.1811
Infiltration	1.9	[1.83–2.06]	**< 0.001**	1.0	[0.88–1.03]	0.2019
Cardiomegaly	1.0	[0.86–1.08]	0.5164	1.1	[0.92–1.3]	0.2914
Consolidation	4.1	[3.54–4.79]	**< 0.001**	0.9	[0.75–1.12]	0.3824
Hernia	0.7	[0.5–1.01]	0.0568	0.9	[0.51–1.51]	0.6361
Fibrosis	0.8	[0.65–0.87]	**0.0002**	1.0	[0.79–1.18]	0.7361
Mass	1.4	[1.23–1.5]	**< 0.001**	1.0	[0.86–1.11]	0.7478
Effusion	4.0	[3.65–4.34]	**< 0.001**	1.0	[0.9–1.14]	0.8272
Pleural thickening	1.3	[1.14–1.47]	**0.0001**	1.0	[0.86–1.2]	0.8349
Pneumonia	2.7	[2.15–3.47]	**< 0.001**	1.0	[0.71–1.34]	0.8605

Follow-up was defined using metadata as a proxy outcome and should be interpreted cautiously. Labels were derived from NLP and are subject to misclassification, particularly for subtle or rare abnormalities. Small sample sizes for some findings (e.g., hernia, fibrosis) may limit estimate stability. “No Finding” served as the reference category and was not included as a predictor; therefore, no interaction term was estimated. Results are exploratory and should not be interpreted as causal evidence or definitive clinical recommendations. Main model: logit Pr(FU = 1) = β₀ + Σ(β_k·Finding_k) + β_sex·Sex.

Interaction model: logit Pr(FU = 1) = β₀ + Σ(β_k·Finding_k) + β_sex·Sex + Σ(γ_k·Finding_k×Sex)..

Sensitivity analyses confirmed robustness of associations across alternative follow-up windows (30, 60, and 180 days). False discovery rate (FDR) adjustment attenuated the significance of some findings but did not alter the main patterns (Supplementary Tables X–Z). Average marginal effects and variance inflation factors are provided in the Supplementary Material (Supplementary Figure A, Supplementary Table W). Supplementary Figure B shows the number of chest radiographs per patient per day; some values (> 4 per day, or fractional) likely reflect metadata artifacts or multiple series being counted separately and were retained as provided in the dataset.

**Fig. 2:**
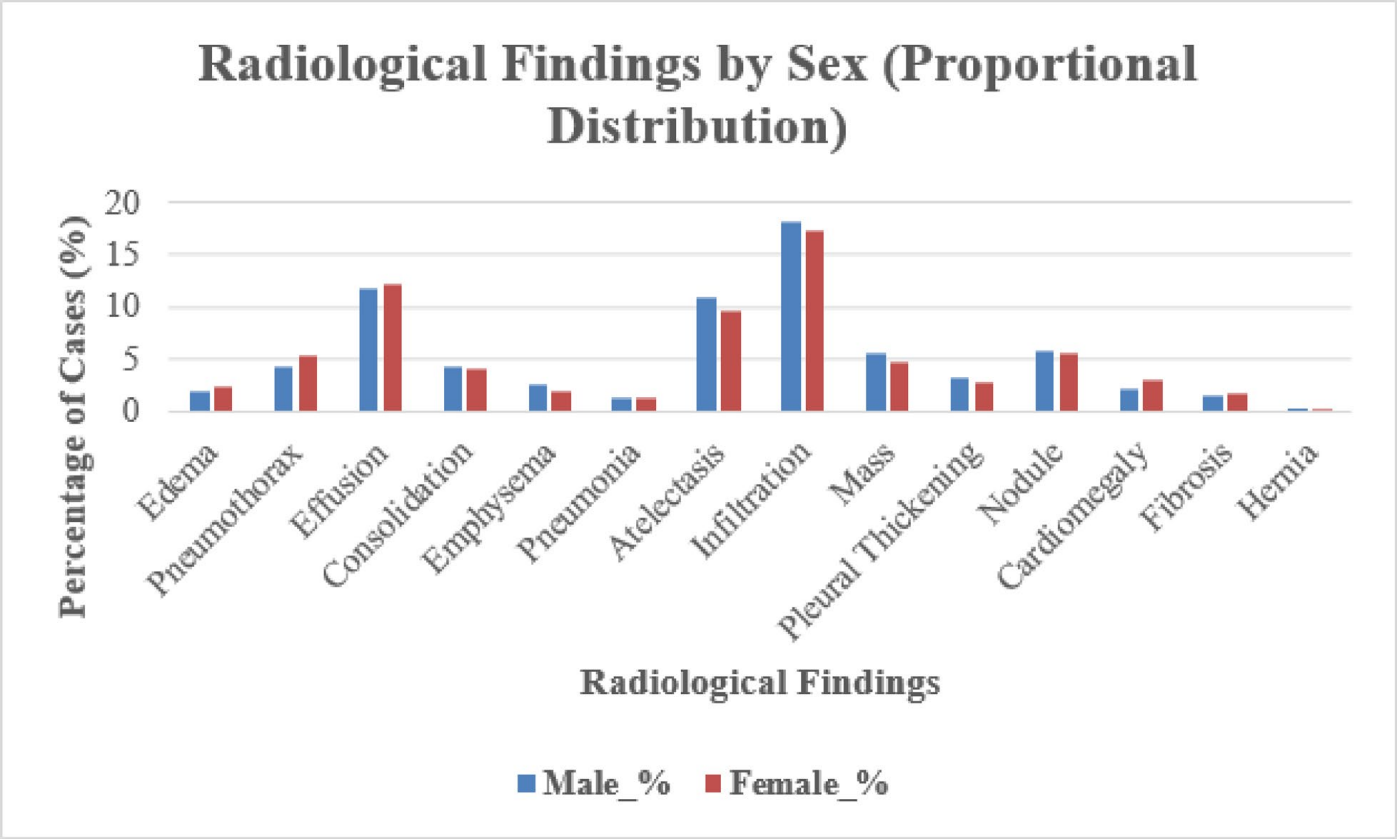
Proportional distribution of radiological findings by sex. Bars show the percentage of male and female radiographs annotated with each abnormality, using distinct grayscale fills/patterns for interpretability. Infiltration, pleural effusion, and atelectasis were the most frequent findings in both sexes, with higher absolute numbers in males, reflecting the dataset distribution.

**Fig. 3:**
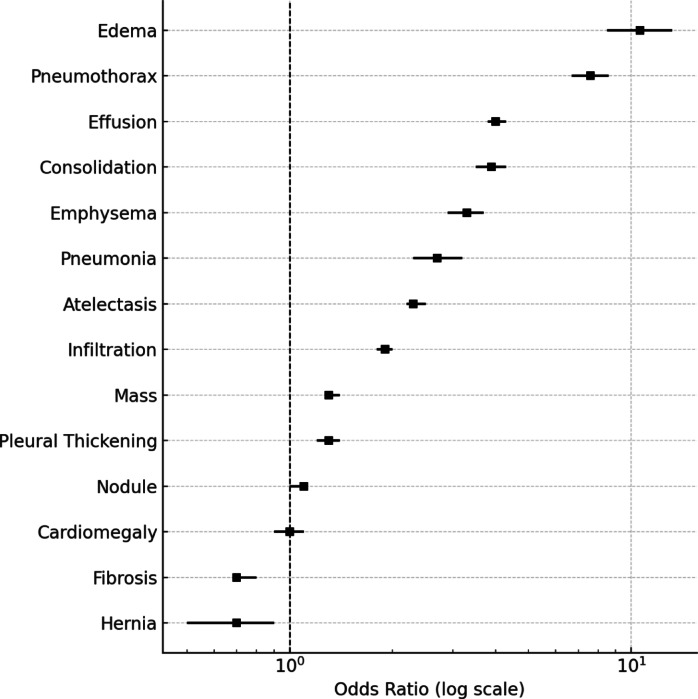
Coefficient plot of odds ratios (ORs) for radiological findings in relation to proxy follow-up. Effect estimates (black squares) with 95% confidence intervals (horizontal lines) were derived from multivariable logistic regression adjusted for sex. The x-axis is logarithmic; the dashed vertical line at OR = 1 indicates no association. Pulmonary edema, pneumothorax, pleural effusion, consolidation, and emphysema were associated with higher odds of follow-up, while fibrosis and diaphragmatic hernia were associated with lower odds.

Figure 2 presents the distribution of radiological findings by sex. Infiltration, effusion, and atelectasis were the most frequent abnormalities in both sexes, with higher absolute case numbers among male patients. Figure 3 displays a coefficient plot summarizing odds ratios across findings on a logarithmic scale.

## Discussion

In this large retrospective analysis of chest radiographs from the ChestX-ray14 dataset, we identified notable associations between specific radiological findings and the likelihood of subsequent proxy follow-up. Pulmonary edema, pneumothorax, and pleural effusion showed the strongest associations, whereas less acute findings such as fibrosis or diaphragmatic hernia were associated with markedly lower follow-up rates. These patterns align with the clinical relevance of the conditions: pulmonary edema reflects decompensated heart failure and respiratory compromise, pneumothorax and effusion typically require urgent management, while fibrosis or hernia often represent chronic or incidental abnormalities.

Our results extend prior research on the diagnostic utility of chest radiography^11^ by quantifying the proxy likelihood of follow-up across findings in a large, publicly available dataset. Pulmonary edema demonstrated the highest odds ratios across both sexes, underlining its role in acute care pathways^12^. Pneumothorax and effusion were also strongly predictive of follow-up, consistent with their acute therapeutic implications^13^. By contrast, findings such as cardiomegaly or fibrosis were weak or inversely associated with follow-up, suggesting either lower perceived urgency or limitations in automated label detection.

Sex-specific analyses revealed statistically significant but modest differences, with pneumothorax more predictive of follow-up in males, and atelectasis and emphysema showing slightly stronger associations in females. These variations are not clinically directive, but they raise exploratory questions about potential biological differences, differential symptom presentation, or variation in provider decision-making. Prior work has highlighted sex-based differences in triage and provider response^14,15^ as well as algorithmic bias in chest radiograph AI applications^16–19^, which may contribute to the observed patterns. However, causal inference cannot be drawn from this analysis.

Sensitivity analyses confirmed the robustness of the main findings across alternative follow-up windows, clustering approaches, and FDR correction. Nonetheless, odds ratios provide relative rather than absolute measures of risk, and marginal probability estimates would improve interpretability for clinical contexts. Future studies should therefore combine odds ratios with absolute effect measures and incorporate calibration metrics to better assess predictive relevance [20].

Taken together, our findings illustrate how radiology-derived metadata can signal meaningful variation in follow-up likelihood while also underscoring the limits of such proxies. The contribution of this work lies in demonstrating that certain findings, particularly pulmonary edema and pneumothorax, consistently align with higher follow-up activity even under proxy definitions. At the same time, the weaker or paradoxical associations for chronic or incidental findings highlight the importance of validating metadata-based research in clinically enriched, multi-institutional datasets.

Overall, this study provides descriptive, hypothesis-generating signals on how chest radiograph findings relate to subsequent activity. The results should not be interpreted as causal evidence or clinical recommendations, but rather as a foundation for future work that links imaging findings with validated electronic health record outcomes and richer clinical covariates.

## Limitations

This study has several limitations. First, the ChestX-ray14 dataset originates from a single tertiary care center and covers a historical period (1992–2015), which may restrict generalizability to contemporary, multi-institutional settings. Second, radiographic labels were generated automatically using a natural language processing (NLP) pipeline applied to reports. Label accuracy varies substantially across findings (e.g., higher for pneumothorax, lower for infiltration), introducing the risk of misclassification bias that may affect odds ratios in a condition-specific manner. Third, the outcome was a metadata-derived proxy rather than a validated measure of clinical follow-up, and may therefore capture both clinical and non-clinical events. Fourth, severity grading was unavailable (e.g., small vs. tension pneumothorax), and acute and chronic abnormalities were analyzed together without distinction. For example, acute findings such as consolidation or pneumothorax may trigger urgent follow-up, whereas chronic conditions such as emphysema or fibrosis may be scheduled independently of the index radiograph. In addition, key covariates such as age, comorbidities, and care setting were not included, limiting interpretability, introducing residual confounding, and preventing assessment of contextual factors. One exploratory proxy analysis using the number of same-day radiographs was performed (Supplementary Figure B), but formal negative-control analyses were not feasible. Furthermore, repeated measures at the patient level were not explicitly modeled, which may violate independence assumptions in regression analyses. Finally, calibration and discrimination analyses (e.g., ROC-AUC, Hosmer–Lemeshow) were not conducted, as the study was designed for exploratory estimation of associations rather than predictive modeling. Taken together, these limitations restrict interpretability, preclude causal inference, and limit clinical applicability. The findings should therefore be regarded strictly as descriptive, hypothesis-generating signals rather than clinically directive evidence.

## Conclusion

These associations were exploratory, and while some sex-specific differences were observed, they were modest and not clinically directive. Because follow-up was derived from metadata rather than validated clinical endpoints, the results must be interpreted with caution and regarded strictly as hypothesis-generating rather than confirmatory. The observed associations may reflect true clinical practice, dataset artifacts, or residual confounding rather than robust causal effects. Future studies linking radiographic findings to electronic health records with richer clinical, demographic, and severity-related data, and conducted across multi-institutional settings, will be essential to clarify these signals. Within these constraints, the present study provides descriptive insights that may guide the design of clinically enriched and hypothesis-driven research.

## Supplementary Information

Below is the link to the electronic supplementary material.

Supplementary Material 1

Supplementary Material 2

Supplementary Material 3

Supplementary Material 4

Supplementary Material 5

Supplementary Material 6

## Data Availability

The NIH ChestX-ray14 dataset used in this study is publicly accessible via the NIH repository: https://nihcc.app.box.com/v/ChestXray-NIHCC (accessed on 7 October 2025).

## Abbreviations

AI: Artificial intelligence
CI: Confidence interval
COVID-19: Coronavirus disease 2019
FDR: False discovery rate
NIH: National Institutes of Health
NLP: Natural language processing
OR: Odds ratio
VIF: Variance inflation factor

## Declarations

### STROBE compliance

This study adheres to the STROBE reporting guideline; the completed STROBE checklist is provided as Supplementary File S1.
