# Supplementary material for: Exploratory associations between radiographic findings and metadata-derived proxies of 90-day follow-up in 112,120 ChestX-ray14 radiographs
Source: Sci Rep. 2025 Dec 9;15:43495. doi: 10.1038/s41598-025-31885-3 (PMC12696044; doi:10.1038/s41598-025-31885-3)
Supplement: Supplementary file 2 — Supplementary Material 2 [file 41598_2025_31885_MOESM2_ESM.docx]

**Supplementary Table W. Multicollinearity assessment (Variance Inflation Factors)**

| **Predictor** | **VIF Value** |
| --- | --- |
| Edema | 1.04 |
| Pneumothorax | 1.04 |
| Effusion | 1.08 |
| Consolidation | 1.03 |
| Emphysema | 1.03 |
| Pneumonia | 1.04 |
| Atelectasis | 1.05 |
| Infiltration | 1.03 |
| Mass | 1.02 |
| Pleural Thickening | 1.02 |
| Nodule | 1.01 |
| Cardiomegaly | 1.02 |
| Fibrosis | 1.00 |
| Hernia | 1.00 |
| Sex | 1.00 |

**Note:** All VIF values were close to 1, indicating no relevant multicollinearity among predictors.
