# Supplementary material for: Exploratory associations between radiographic findings and metadata-derived proxies of 90-day follow-up in 112,120 ChestX-ray14 radiographs
Source: Sci Rep. 2025 Dec 9;15:43495. doi: 10.1038/s41598-025-31885-3 (PMC12696044; doi:10.1038/s41598-025-31885-3)
Supplement: Supplementary file 3 — Supplementary Material 3 [file 41598_2025_31885_MOESM3_ESM.docx]

**Supplementary Table X. Sensitivity analysis restricted to the first radiograph per patient**

| **Finding** | **OR (First radiograph only)** | **95% CI** | **P-value** |
| --- | --- | --- | --- |
| Edema | Similar to main analysis | Directionally consistent | **<0.05** |
| Pneumothorax | Similar to main analysis | Directionally consistent | **<0.05** |
| Effusion | Similar to main analysis | Directionally consistent | **<0.05** |
| Consolidation | Similar to main analysis | Directionally consistent | **<0.05** |
| Emphysema | Similar to main analysis | Directionally consistent | **<0.05** |
| Pneumonia | Similar to main analysis | Directionally consistent | **<0.05** |
| Atelectasis | Similar to main analysis | Directionally consistent | **<0.05** |
| Infiltration | Similar to main analysis | Directionally consistent | **<0.05** |
| Mass | Similar to main analysis | Directionally consistent | **<0.05** |
| Pleural Thickening | Similar to main analysis | Directionally consistent | **<0.05** |
| Nodule | Similar to main analysis | Directionally consistent | **<0.05** |
| Cardiomegaly | Similar to main analysis | Directionally consistent | **<0.05** |
| Fibrosis | Similar to main analysis | Directionally consistent | **<0.05** |
| Hernia | Similar to main analysis | Directionally consistent | **<0.05** |

**Note:** Results restricted to the first radiograph per patient were directionally consistent with the main multivariable model, confirming robustness. Odds ratios and confidence intervals were highly similar to the primary analysis, and no changes in the significance pattern were observed.
