# Supplementary material for: Exploratory associations between radiographic findings and metadata-derived proxies of 90-day follow-up in 112,120 ChestX-ray14 radiographs
Source: Sci Rep. 2025 Dec 9;15:43495. doi: 10.1038/s41598-025-31885-3 (PMC12696044; doi:10.1038/s41598-025-31885-3)
Supplement: Supplementary file 4 — Supplementary Material 4 [file 41598_2025_31885_MOESM4_ESM.docx]

**Supplementary Table Y. Multiple testing adjustment using Benjamini–Hochberg FDR**

| **Finding** | **OR** | **95% CI** | **Nominal P-value** | **FDR-adjusted P-value** |
| --- | --- | --- | --- | --- |
| Edema | 10.6 | 8.5–13.2 | **<0.001** | **<0.05** |
| Pneumothorax | 7.6 | 6.7–8.6 | **<0.001** | **<0.05** |
| Effusion | 4.0 | 3.8–4.3 | **<0.001** | **<0.05** |
| Consolidation | 3.9 | 3.5–4.3 | **<0.001** | **<0.05** |
| Emphysema | 3.3 | 2.9–3.7 | **<0.001** | **<0.05** |
| Pneumonia | 2.7 | 2.3–3.2 | **<0.001** | **<0.05** |
| Atelectasis | 2.3 | 2.2–2.5 | **<0.001** | **<0.05** |
| Infiltration | 1.9 | 1.8–2.0 | **<0.001** | **<0.05** |
| Mass | 1.3 | 1.3–1.4 | **<0.001** | **<0.05** |
| Pleural Thickening | 1.3 | 1.2–1.4 | **<0.001** | **<0.05** |
| Nodule | 1.1 | 1.0–1.1 | **0.0357** | >0.05 (n.s.) |
| Cardiomegaly | 1.0 | 0.9–1.1 | 0.9304 | >0.05 (n.s.) |
| Fibrosis | 0.7 | 0.7–0.8 | **<0.001** | **<0.05** |
| Hernia | 0.7 | 0.5–0.9 | **0.0037** | **<0.05** |

**Footnote:** Nominal P-values are shown alongside Benjamini–Hochberg false discovery rate (FDR) adjusted P-values. After FDR correction, most associations (e.g., edema, pneumothorax, effusion, consolidation, emphysema, pneumonia, atelectasis, infiltration, mass, pleural thickening, fibrosis) remained statistically significant. However, low-prevalence findings such as nodule and hernia, as well as cardiomegaly, did not survive correction (n.s.). Results are exploratory and should be interpreted with caution.
