# Supplementary material for: Exploratory associations between radiographic findings and metadata-derived proxies of 90-day follow-up in 112,120 ChestX-ray14 radiographs
Source: Sci Rep. 2025 Dec 9;15:43495. doi: 10.1038/s41598-025-31885-3 (PMC12696044; doi:10.1038/s41598-025-31885-3)

**Supplementary Figure A.** Average marginal effects (AME) of radiological findings on the probability of follow-up. Bars represent absolute percentage-point changes with 95% confidence intervals. Edema, pneumothorax, and pleural effusion showed the largest absolute increases in predicted follow-up probability.


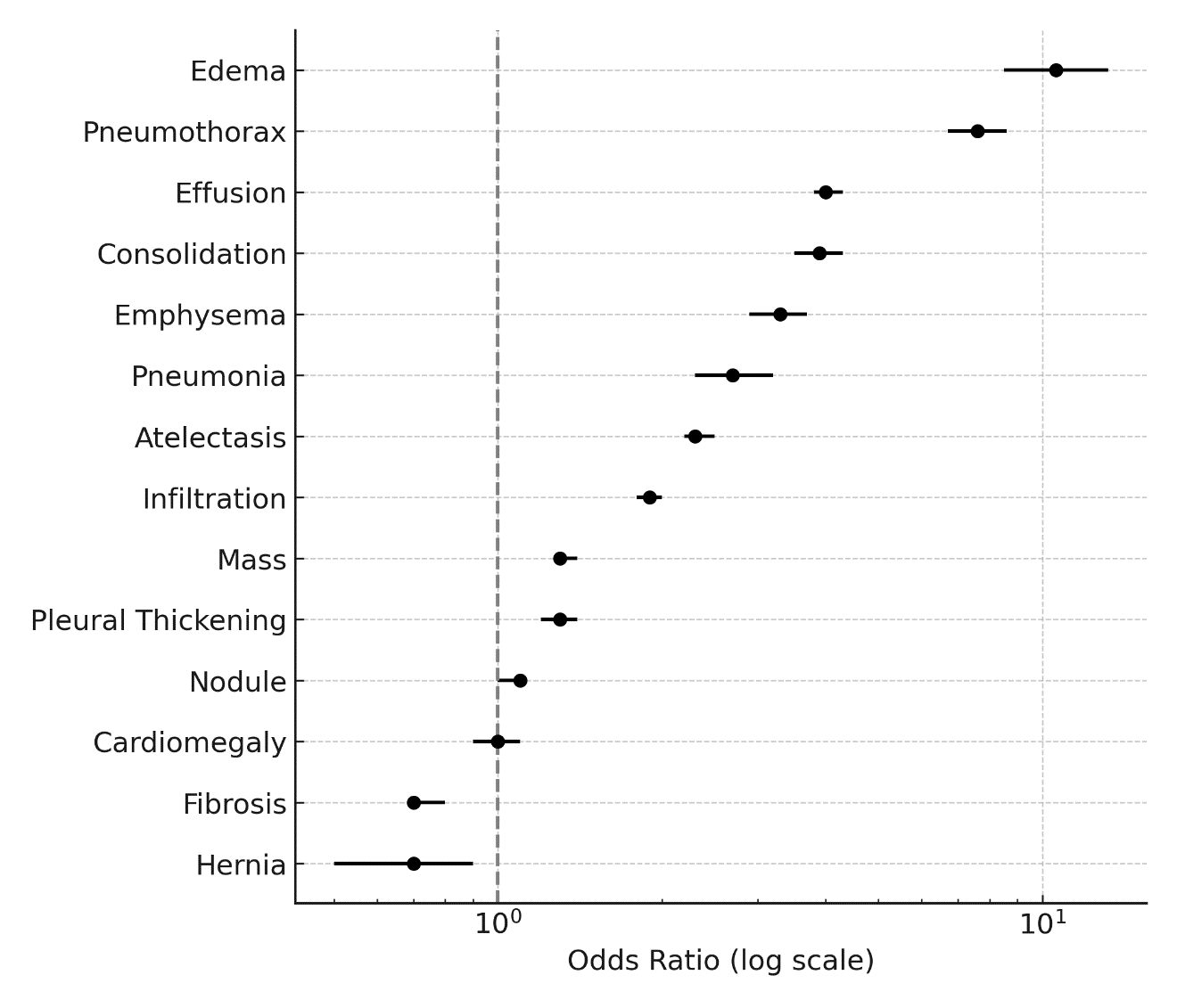

Supplement: Supplementary file 5 — Supplementary Material 5 [file 41598_2025_31885_MOESM5_ESM.docx]
