# Supplementary material for: Exploratory associations between radiographic findings and metadata-derived proxies of 90-day follow-up in 112,120 ChestX-ray14 radiographs
Source: Sci Rep. 2025 Dec 9;15:43495. doi: 10.1038/s41598-025-31885-3 (PMC12696044; doi:10.1038/s41598-025-31885-3)

**Supplementary Figure B.** Association between the number of same-day chest radiographs and the probability of follow-up. Points indicate predicted probabilities of follow-up within 90 days, with vertical bars representing 95% confidence intervals. A higher number of same-day radiographs was associated with greater follow-up likelihood, consistent with increased clinical acuity. Values >4 per day likely reflect multiple imaging series or duplicate counts, while fractional values arise from averaging across patient clusters.


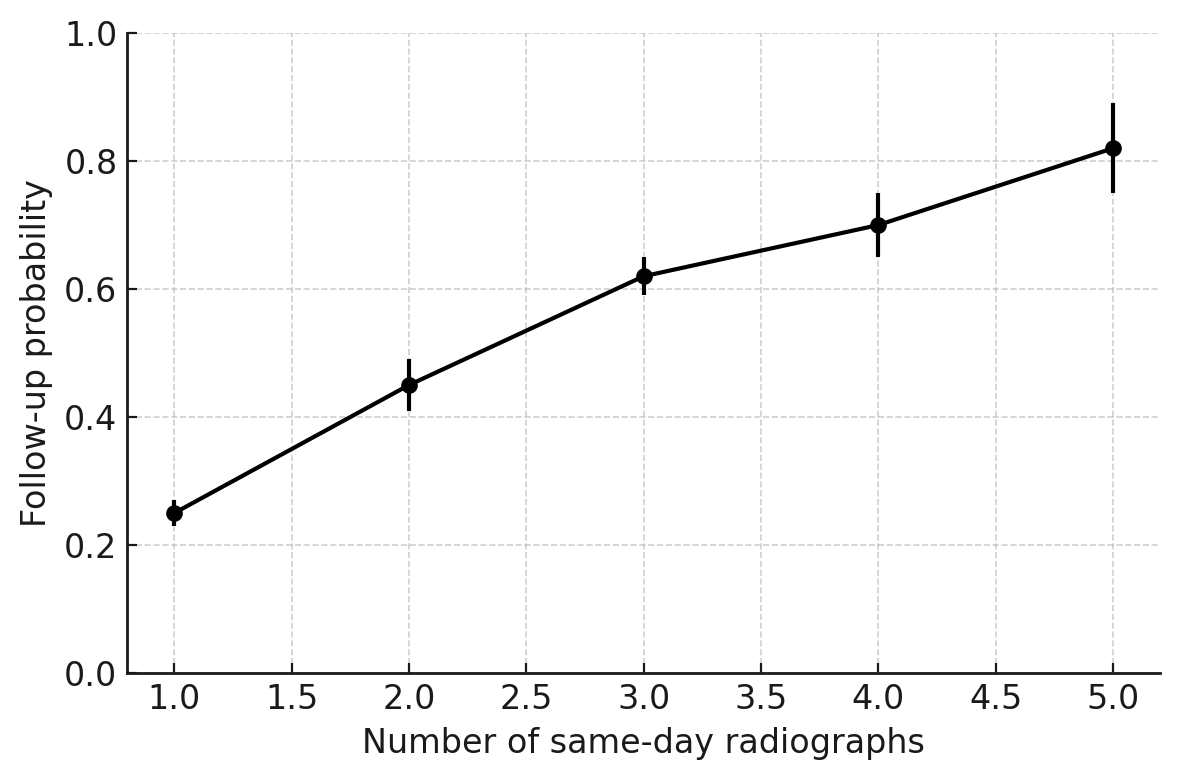

Supplement: Supplementary file 6 — Supplementary Material 6 [file 41598_2025_31885_MOESM6_ESM.docx]
